# Comparative Transcriptome Analysis of Two Cucumber Cultivars with Different Sensitivity to Cucumber Mosaic Virus Infection

**DOI:** 10.3390/pathogens9020145

**Published:** 2020-02-21

**Authors:** Zdeno Šubr, Lukáš Predajňa, Katarína Šoltys, Boris Bokor, Jaroslav Budiš, Miroslav Glasa

**Affiliations:** 1 Institute of Virology, Biomedical Research Center of the Slovak Academy of Sciences, Dúbravská cesta 9, 845 05 Bratislava, Slovakia; Lukas.Predajna@savba.sk (L.P.); Miroslav.Glasa@savba.sk (M.G.); 2 Comenius University Science Park, Comenius University in Bratislava, Ilkovičova 8, 841 04 Bratislava, Slovakia; katarina.soltys@gmail.com (K.Š.); boris.bokor@gmail.com (B.B.); jaroslav.budis@cvtisr.sk (J.B.); 3 Department of Microbiology and Virology, Comenius University in Bratislava, Ilkovičova 6, 841 04 Bratislava, Slovakia; 4 Department of Molecular Biology, Comenius University in Bratislava, Ilkovičova 6, 841 04 Bratislava, Slovakia; 5 Geneton Ltd., Ilkovičova 8, 841 04 Bratislava, Slovakia; 6 Slovak Centre of Scientific and Technical Information, Lamačská cesta 7315/8A, 811 04 Bratislava, Slovakia; 7 Faculty of Natural Sciences, University of Ss. Cyril and Methodius, Nám. J. Herdu 2, 91701 Trnava, Slovakia

**Keywords:** CMV, gene expression, compatible infection, resistance, NGS

## Abstract

Cucumber mosaic virus (CMV), with extremely broad host range including both monocots and dicots around the world, belongs to most important viral crop threats. Either natural or genetically constructed sources of resistance are being intensively investigated; for this purpose, exhaustive knowledge of molecular virus-host interaction during compatible and incompatible infection is required. New technologies and computer-based “omics” on various levels contribute markedly to this topic. In this work, two cucumber cultivars with different response to CMV challenge were tested, i.e., sensitive cv. Vanda and resistant cv. Heliana. The transcriptomes were prepared from both cultivars at 18 days after CMV or mock inoculation. Subsequently, four independent comparative analyses of obtained data were performed, viz. mock- and CMV-inoculated samples within each cultivar, samples from mock-inoculated cultivars to each other and samples from virus-inoculated cultivars to each other. A detailed picture of CMV-influenced genes, as well as constitutive differences in cultivar-specific gene expression was obtained. The compatible CMV infection of cv. Vanda caused downregulation of genes involved in photosynthesis, and induction of genes connected with protein production and modification, as well as components of signaling pathways. CMV challenge caused practically no change in the transcription profile of the cv. Heliana. The main differences between constitutive transcription activity of the two cultivars relied in the expression of genes responsible for methylation, phosphorylation, cell wall organization and carbohydrate metabolism (prevailing in cv. Heliana), or chromosome condensation and glucan biosynthesis (prevailing in cv. Vanda). Involvement of several genes in the resistant cucumber phenotype was predicted; this can be after biological confirmation potentially applied in breeding programs for virus-resistant crops.

## 1. Introduction

Cucumber mosaic virus (CMV) is the type member of the genus *Cucumovirus* (family *Bromoviridae*). Each of its tripartite (+)ssRNA genome segments is separately encapsidated in non-enveloped virions with icosahedral symmetry. CMV is world-wide spread and its known natural host range is extremely broad [1]. CMV infects over 1200 plant species in more than 100 families of monocots and dicots [2]. Many host species are economically important vegetable crops where the CMV infection causes severe damage. Natural sources of resistance are continually sought and potentially resistant plants are constructed using targeted genetic manipulations as well [3,4,5]. Virus-host interaction represents a complex process where many plant factors are included. Virus infection influences expression of various plant genes to redirect biosynthetic pathways in favor of viral progeny production. On the other hand, host defense genes try to eliminate the infection. Mutual influence of these processes on several levels results in both virus-beneficial and unintended consequences of infection for the host plant [6].

CMV is a relatively intensively studied virus also regarding interactions with host organisms [7,8]. Most data have been obtained from the model plant *Arabidopsis thaliana*, where several coordinated mechanisms of defense response have been discovered, including RNA-mediated gene silencing, salicylic acid (SA)-dependent and independent regulation patterns and expression of resistance genes [9]. Analysis of CMV resistome in *Arabidopsis* showed mainly directed regulation of kinases/phosphatases, of protein degradation factors, transcription regulators and many short polypeptides of unknown function [10].

Recently we tested several cucumber cultivars for their sensitivity to various viruses and found an appropriate virus-host system for detailed study of interactions during viral infection [11]. The cv. Vanda appeared to be very sensitive to CMV infection, showing severe symptoms, the high virus concentration detected in the plants immunochemically, as well as by RT-PCR. On the other hand, the cv. Heliana showed no symptoms under identical experimental conditions and CMV could be detected neither by RT-PCR, nor by immunoblotting in any inoculated plant. In this cultivar obviously extreme resistance (immunity) was manifested. In this work we show the results of comparative transcriptomics analysis of sensitive and resistant cultivars before and after exposition to the CMV challenge.

## 2. Results and Discussion

**Figure 1:**
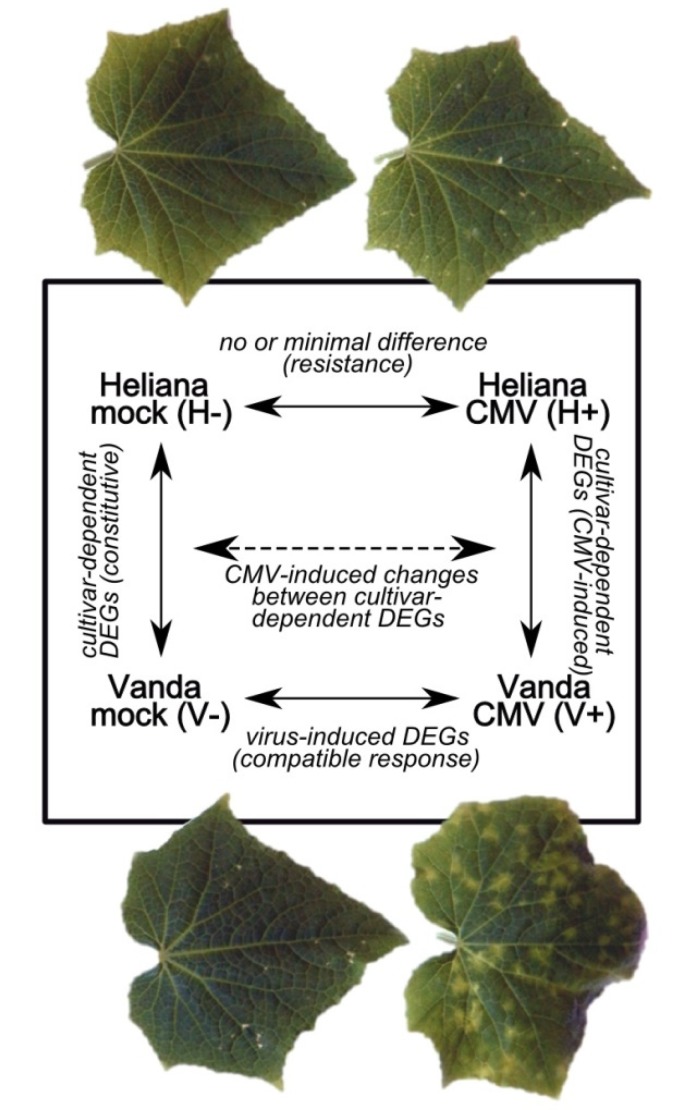
Scheme of transcriptome comparisons performed in this work.

Individual samples were labeled as H- (Heliana mock), H+ (Heliana/CMV), V- (Vanda mock) and V+ (Vanda/CMV). Four independent comparisons were performed. Transcriptomes of mock-inoculated and CMV-inoculated plants in frame of each cultivar (H-/H+, V-/V+) were compared; furthermore, gene expression analysis within the mock-inoculated cultivars (H-/V-) and within virus-inoculated cultivars (H+/V+) was determined, too. Each of the four comparisons provides different evidence (Figure 1) and their combination enables a complex evaluation of cucumber reaction to the middle-late state of CMV infection. Particular comparisons fairly differed by the number of detected significant differentially expressed genes (DEGs) (Table 1). The most divergent gene expression was detected in comparative analyses V-/V+ and H+/V+, total, the number of up- and down-regulated genes was similar in these analyses. The lowest number of significant DEGs showed the transcriptomes of mock- and CMV-inoculated (but also not infected) cv. Heliana. Mutual comparison of mock-inoculated cultivars showed approximately four times less DEGs as CMV-inoculated cultivars. In CMV-free cv. Vanda totally app. two-times less genes as in cv. Heliana was expressed, however, upon inoculation slightly more DEGs were found in cv. Vanda. These quantitative results were in accord with the reactivity of respective cultivars to the viral infection.

### 2.1. Gene Ontology Categorization of DEGs

Only minimal differences were found between the control and CMV-inoculated cv. Heliana (H-/H+), which did not enable quantitative evaluation of functionally annotated DEGs.

Comparison of mock- and CMV-inoculated cv. Vanda (V-/V+) showed negative influence of CMV on activities connected with photosynthesis (GO:0009535, chloroplast thylakoid membrane; GO:0009941, chloroplast envelope; GO:0009570, chloroplast stroma; GO:0009507, chloroplast; GO:0009523, photosystem II; GO:0016168, chlorophyll binding; GO:0018298, protein-chromophore linkage; GO:0015979, photosynthesis; GO:0009522, photosystem I; GO:0045156, electron transporter, transferring electrons within the cyclic electron transport pathway of photosynthesis activity; GO:0048038, quinone binding), protein modification (GO:0006470, protein dephosphorylation), polysaccharide metabolism (GO:2001070, starch binding; GO:0030244, cellulose biosynthetic process; GO:0010411, xyloglucan metabolic process), redox balance (GO:0015035, protein disulfide oxidoreductase activity; GO:0051213, dioxygenase activity; GO:0051287, NAD binding), stress signal transduction (GO:0009409, response to cold; GO:0009611, response to wounding; GO:0009414, response to water deprivation; GO:0009734, auxin-activated signaling pathway; GO:0006662, glycerol ether metabolic process; GO:0005992, trehalose biosynthetic process; GO:0009734, auxin-activated signaling pathway; GO:0006855, drug transmembrane transport) or lipid metabolic process (GO:0006629).

On the other hand, several processes were stimulated during infection, including proteosynthesis, maturation and degradation of proteins (GO:0005789, endoplasmic reticulum membrane; GO:0005788, endoplasmic reticulum lumen; GO:0003735, structural constituent of ribosome; GO:0006412, translation; GO:0006886, intracellular protein transport; GO:0004298, threonine-type endopeptidase activity; GO:0051082, unfolded protein binding; GO:0006486, protein glycosylation; GO:0006886, intracellular protein transport; GO:0000502, proteasome complex; GO:0006457, protein folding), transport (GO:0042626, ATPase activity, coupled to transmembrane movement of substances; GO:0016192, vesicle-mediated transport; GO:0006811, ion transport) and some other activities (GO:0042744, hydrogen peroxide catabolic process; GO:0043531, ADP binding; GO:0030246, carbohydrate binding; GO:0005516, calmodulin binding; GO:0031047, gene silencing by RNA; GO:0004568, chitinase activity; GO:0035235, ionotropic glutamate receptor signaling pathway).

**Figure 2:**
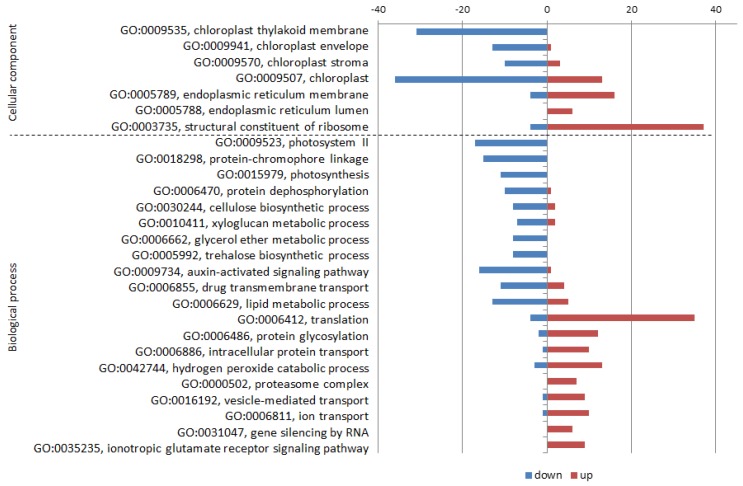
GO categories and number of relevant DEGs substantially repressed or induced by cucumber mosaic virus (CMV) infection in the cv. Vanda.

GO categories with most expressive differences in expression levels are depicted in the Figure 2. Generally, this picture reflected that the metabolic processes were redirected to produce new virus particles. Virus replication and virion maturation require enhanced proteosynthesis while transformation of solar energy by photosynthesis is reduced by reason of lower number and quality of chloroplasts. In later stage of the compatible infection (33 dpi) in the same virus-host system, the abundance of proteins involved in translation decreased, as showed the proteomic analysis [12]. In the late phase the supplied energy obviously cannot suffice both function and repair of the overloading proteosynthetic apparatus which undergo consecutive degradation.

Transcriptome analysis of both CMV-free cultivars (H-/V-) revealed markedly predominating GO groups in the resistant cultivar Heliana, connected with regulation on various levels (GO:0032259, methylation; GO:0016301, kinase activity; GO:0003700, DNA binding transcription factor activity), metabolism (GO:0030246, carbohydrate binding; GO:0016747, transferase activity, transferring acyl groups other than amino-acyl groups; GO:0004553, hydrolase activity, hydrolyzing O-glycosyl compounds), redox processes (GO:0050660, flavin adenine dinucleotide binding; GO:0016614, oxidoreductase activity, acting on CH-OH group of donors) and iron transport (GO:0020037, heme binding; GO:0005506, iron ion binding). 

In the sensitive cv. Vanda groups targeting regulation and degradation of DNA (GO:0000737, DNA catabolic process, endonucleolytic; GO:0042138, meiotic DNA double-strand break formation; GO:0007076, mitotic chromosome condensation; GO:0016889, endodeoxyribonuclease activity, producing 3′-phosphomonoesters) and metabolism of fungal cell wall (GO:0006075, (1->3)-beta-D-glucan biosynthetic process) prevailed.

**Figure 3:**
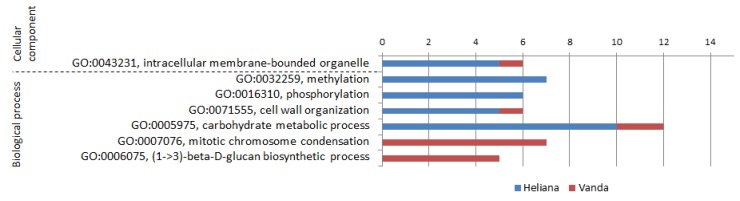
GO categories and number of relevant DEGs substantially differently expressed in CMV-free cultivars Heliana and Vanda.

As summarized in Figure 3, the main differences between constitutive transcriptomes of the two cultivars were detected in expression of genes involved in methylation, phosphorylation, cell wall organization and carbohydrate metabolism (prevailing in cv. Heliana), or chromosome condensation and glucan biosynthesis (prevailing in cv. Vanda).

Comparison of the cultivars after CMV inoculation (H+/V+) was similar as differences between mock- and CMV-inoculated sensitive cultivar. In cv. Heliana prevailing gene groups were connected with photosynthesis (GO:0009535, chloroplast thylakoid membrane; GO:0009570, chloroplast stroma), regulation and signal transduction, especially in stress conditions (GO:0005992, trehalose biosynthetic process; GO:0009734, auxin-activated signaling pathway; GO:0000160, phosphorelay signal transduction system; GO:0003951, NAD+ kinase activity; GO:0009611, response to wounding), cell wall synthesis (GO:0030244, cellulose biosynthetic process; GO:0010411, xyloglucan metabolic process) and lipid catabolic process (GO:0016042).

**Figure 4:**
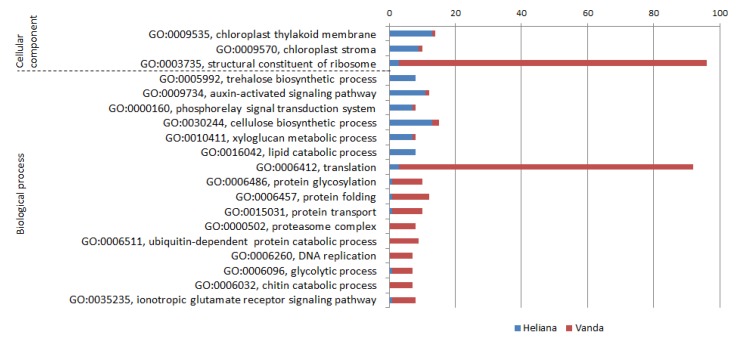
GO categories and number of relevant DEGs substantially differently expressed in CMV-inoculated cultivars Heliana and Vanda.

On the other hand, in cv. Vanda predominating transcripts belonged to groups connected with proteosynthesis, protein modification and degradation (GO:0003735, structural constituent of ribosome; GO:0006412, translation; GO:0006486, protein glycosylation; GO:0006457, protein folding; GO:0015031, protein transport; GO:0051082, unfolded protein binding; GO:0006511, ubiquitin-dependent protein catabolic process; GO:0000502, proteasome complex; GO:0006511, ubiquitin-dependent protein catabolic process; alpha-subunit complex; GO:0004298, threonine-type endopeptidase activity), DNA replication (GO:0006260, DNA replication; GO:0007076, mitotic chromosome condensation) and other activities (GO:0030246, carbohydrate binding; GO:0005516, calmodulin binding; GO:0006096, glycolytic process; GO:0006032, chitin catabolic process; GO:0035235, ionotropic glutamate receptor signaling pathway). GO categories with highest differences between CMV-inoculated cultivars are depicted in the Figure 4.

The strongest differences between cultivars concerning GO groups of both constitutive and virus-induced gene expression were GO:0007076, mitotic chromosome condensation and GO:0006075, (1->3)-beta-D-glucan biosynthetic process (both intensively expressed in cv. Vanda and absent in Heliana), and GO:0032259, methylation (clearly prevailing in cv. Heliana). As DNA methylation is a key process of epigenetic regulation, different expression of genes for relevant enzymes could explain different sensitivity of the cultivars to CMV challenge. Further we describe selected specific genes/proteins which markedly differed in their expression in frame of particular comparisons. The values of samples from biological triplicates were often considerably variable. Therefore, we focused on the significant DEGs, where the standard deviation was lower than 10% of mean value of the triplicates (the numbers in parentheses in the Table 1).

### 2.2. Mock- and CMV-Inoculated cv. Heliana (Comparison H-/H+)

Very low number of DEGs found after CMV inoculation of cv. Heliana is in agreement with the absence of any visible symptoms and zero detected level of the virus in plant tissues. Nine genes were significantly influenced (Table S1), of which only for one the consistent results from triplicates were obtained (Table 1). Approximately twofold decrease of L-ascorbate oxidase (AO) gene expression was observed. This apoplast protein (localized in the cell wall) binding copper ions reduces molecular oxygen to water using ascorbate as electron donor [13]. Influence of AO on the cell growth and negative correlation of its expression with wound healing were found [14,15]. This enzyme obviously participates in general maintenance of redox equilibrium, in signaling pathways and mutualistic interactions between plants and microorganisms [16]. Several authors found correlation between reduced level or activity of AO and plant tolerance to various stress types [17,18]. Ascorbic acid is an important antioxidant protecting plants from oxidative stress induced by pathogen attack and AO is a key regulator of the cellular ascorbate level balance [19]. We can only speculate if decreased expression of AO and CMV challenge were causally connected in this case. Recently, an interaction of AO with CMV movement protein (MP) in a compatible infection of cucumber was detected and experimental knock-out of the AO gene lead to decreased virus accumulation in systemic infected leaves. The results indicated importance of MP-AO interaction for the virus transport in the early infection state [20]. On the other hand, experimental AO degradation negatively influenced the rice resistance to the rice strip virus, probably due to decrease of reactive oxygen species accumulation [21].

Regarding the group of differentially expressed genes with higher deviation among samples triplicates, the biggest difference was detected for patatin (decreased expression after inoculation). The group of patatins and patatin-like proteins (PLPs) includes storage glycoproteins with enzyme activities focused on lipid metabolism (esterase, acyl transferase, lipidacyl hydrolase, phospholipase) [22]. Their role at Ca-dependent signal transduction in plants has been presumed [23]. Overproduction of PLPs has been correlated with biotic and abiotic stress including viral infection [24,25,26,27]. On the other hand, Cheng et al. [28] found a negative influence of PLP on resistance to fungal attack of *Nicotiana attenuata*. Our results (3-fold fall of expression in cv. Heliana versus 29-fold depression in the sensitive cv. Vanda) also supported possible involvement of patatin in plant stress pathways.

### 2.3. Mock- and CMV-Inoculated cv. Vanda (Comparison V-/V+)

In the cv. Vanda CMV infection was manifested by intensive leaf symptoms and high virus concentration was confirmed immunochemically. Concerning V-/V+ DEGs, we detected comparable number of induced and repressed genes (Table S2).

#### 2.3.1. Down-Regulation by CMV Infection

The most visible change in gene expression of the down-regulated genes (over 6-fold) was detected for the MIZU-KUSSEI 1 (MIZ1), recently discovered factor influencing the root hydrotropism [29]. MIZ1 regulates negatively the cytokinin sensitivity on root development and is important for development of lateral roots through auxin level in *Arabidopsis* [30].

Expression of inositol-tetrakisphosphate kinase (ITPK1) was 3.6-times reduced. This enzyme takes part in phosphorylation/dephosphorylation of inositol phosphates, the precursors of inositol pyrophosphates—universal energetic signal molecules [31]. IPTK1 may play regulation role in various processes. Contrary to our result, rather its stress-induction has been documented. In geminivirus-infected tomato its level increased twice [32]. IPTK1 induction has been recorded also during tobacco flooding stress [33]. Repression of this enzyme during induction of dormancy in peach has been interpreted as blocking the phosphorylation signals and ATP production connected with lowered photosynthesis level [34]. Such explanation correlates also with photosynthesis drop during CMV infection of the compatible host.

The proteins with pentatricopeptid repetitions (PPRs) are massively represented in plant proteomes. Their expansion due to retrotransposition is presumed [35]. They regulate expression of mitochondrial and plastid genes by various mechanisms and their combined actions dramatically affect the biogenesis and function of organelles in plant cells [36]. Expression of several PPRs was 1.3–3-times repressed by the CMV infection in cv. Vanda, probably influencing chloroplast formation and chlorotic symptom production.

Over 3-fold down-regulation of cytochrome P450 could influence many processes in which this monooxygenase take part, including growth, differentiation, organogenesis and stress reactions [37]. Narusaka et al. [38] found in *Arabidopsis* mostly stress-induction of P450, but several its forms were repressed by some abiotic factors.

The TIFY group proteins are transcription factors involved in regulation of phytohormone-dependent biological processes [39]. A subgroup of them, proteins with jasmonate ZIM-domain (JAZ) repress the jasmonic acid (JA) signalization. These proteins have been induced e.g., by bicarbonate stress in soybean [39], by various types of abiotic and biotic stress in rice [40,41] or *Brassica rapa* [42]. However, particular elements of JA-mediated defense response against cellular pathogens are generally rather negatively influenced by viral infection [43]. Several genes coding for TIFY proteins (TIFY 10a, TIFY 10b-like, TIFY 10c-like) were found to be repressed (1.9–2.5-times lower expression) after CMV infection in the cv. Vanda. TIFY expression has been correlated with some plant infections caused by geminiviruses or reoviruses [41,44], however, no data for CMV or other ssRNA viruses were found in the literature.

#### 2.3.2. Up-Regulation by CMV Infection

The differences between expression levels of virus-induced genes were more expressive (1.5–22.5-fold compared to transcriptome from uninfected plants). Slightly higher expression of ribosomal proteins reflected higher requirement on proteosynthesis in infected tissues.

SPO-11-1 (nearly 15-fold induced) is part of topoisomerase 6 complex, necessary for meiotic recombination [45]. Exposition of plants to pathogen challenge may lead to enhanced somatic recombination, as demonstrated for tobacco mosaic virus (TMV)-infected tobacco [46]. Higher gene rearrangement frequency may by an adaptive advantage in defense against pathogens [47].

Enzymes from the ubiquitous family of glutathion S-transferases (GSTs) are generally involved in detoxification processes in living organisms. Inducibility by various stress factors (often SA-mediated) is typical for GST genes [48]. Concerning plant-virus relation, different correlation between virus level and plant GSTs have been detected. In tobacco plants with hypersensitive reaction to TMV infection the GST concentration transiently dropped just before symptom creation, but thereafter substantially increased [49]. Similarly, GST was induced after lesion development in *Arabidopsis* infected by CMV [50]. During infection by Rice tungro spherical virus induction of GST genes has been found in the resistant rice cultivar. In the susceptible cultivar several of them have been repressed in the early state, however, later they have been induced again [51]. Up-regulation of GST during viral infections has been repeatedly demonstrated [52,53]. It is possible that this enzyme directly supports the replication of some viruses [54]. In the light of these facts our data showing 11-times higher GST expression in the susceptible cucumber cultivar after CMV infection were expectable.

Derlin (6-times up-regulated) is a part of machinery for misfolded protein degradation in the endoplasmic reticulum (ER), although detailed mechanism of its action in plants is not known [55]. Accumulation of misfolded proteins in ER induced derlin expression in rice [56]. At least for some picornaviruses the host derlin is even essential for successful infection [57,58]. Derlin expression has been repressed by bacterial infection of pepper [59], but induced by rhabdovirus in maize [60]. Massive proteosynthesis during viral infection leads to ER stress and following induction of relevant proteins including derlin [61].

Many other detected induced DEGs could be directly or indirectly bound to the virus infection, as they have been positively correlated with plant stress reaction: signal and regulation factors like chloroplast sigma factor-binding protein [62], sterile alpha motif-containing protein [63], resistance protein with TIR-NBS-LRR domain [64], numerous protein kinases, proteases and parts of proteasome [65]. It has been shown, that the 26S proteasome system participates in degradation of TMV MP, specific inhibition experiments, however, have indicated that TMV infection rather benefits from this process [66]. Plant aspartate protease Asp1 interacted with the begomoviral C4 protein in the yeast two-hybrid system [67], which may indicate its activity and intervention during viral infection. The NRT1/PTR family proteins (nitrate/peptide transporters) take part in abscisic acid transport in plant tissues [68].

For some DEGs several forms were detected with different range of change (both up- and down-regulated). It was the case of diacylglycerol kinase, glycerol-3-phosphate dehydrogenase, kinesin-like protein, transcription factor WRKY, protein DETOXIFICATION, RING E3 or ubiquitin transferase.

### 2.4. Mock-Inoculated Cultivars (Comparison H-/V-)

Comparison of the two CMV-free cultivars provides the information about transcriptome differences irrespective of viral infection. It summarizes the list of genes, the presence/expression of which could have preventive (prophylactic) influence on the CMV challenge—potential resistance genes. The fact, that only few genes were influenced by Heliana inoculation confirmed the “non-host“ status of this cultivar as result of constitutive genome expression and implicates expectations to spot the genes responsible for the resistant phenotype.

For 60% from the 53 analyzed DEGs (Table S3), the expression was lower in the cv. Vanda compared to cv. Heliana. The highest detected difference concerned the UBX-domain protein 4 (PUX4), which was not found in Vanda at all. Ubiquitin-regulating region X (UBX) proteins create a group of cofactors of the AAA ATPase Cdc48/p97. It is an ubiquitinated protein-binding chaperon active at degradation of misfolded proteins. The proteins with ubiquitin-regulating region X (UBX) participate in Cdc48/p97-substrate binding, as well as in time and space regulation its activity [69]. It probably substantially contributes to plant immunity by precise control of produced proteins in virus-attacked cells [70]. On the other hand, some authors presume positive influence of the ubiquitin-proteasome system on infection by (+)ssRNA viruses by regulation of concentration of particular viral proteins [71]. Our results are in line with the former scenario, as the absence of PUX4 correlated with the susceptibility to CMV infection. The difference was of constitutive nature, inoculation did not affect the PUX4 expression in neither cultivar. Similar correlation was found also with PUX9 (2.8-fold lower in Vanda), other members of this group showed no significant differences. PUX4 is an eminent candidate gene which may serve as key factor influencing the sensitivity of analyzed cucumber cultivars to the CMV infection.

Another possible candidate is RPM1-interacting protein 4 (RIN4), an essential regulator of plant defense reaction. RPM1 is localized in the cytoplasmic membrane where (by the medium of RIN4) it interacts with bacterial virulent factors and induces the hypersensitive reaction and infection elimination [72]. RIN4 interacts with integrin-like factor NDR1, which mediates the stress signalization and modified stomatal apertures during the pathogen attack [73]. It is still not clear if this system plays a role also at viral infections [74]. RPM1 expression was more than two-fold lower in the sensitive cv. Vanda.

For several glycosyl transferases 1.5–3-times lower expression in cv. Vanda was detected. These enzymes participate in creation and modification of various glycoconjugates. Repression of glycosyl transferase led to decrease of tobacco resistance to TMV [75]. On the other hand, its overproduction increased the resistance to potato virus Y [76]. Majority of *Arabidopsis* glycosyl transferase genes have been induced by infection of several bacterial pathogens [77]. We also detected over 10-fold increase of glycosyl transferase expression in the cv. Vanda due to CMV infection, while in the cv. Heliana its level did not change.

Furthermore, the susceptibility to the infection reflected by other DEGs was in accord with published data, including the nudix hydrolase [78], the lipid-transport protein [79], the resistance protein with NB-ARC domain [80], GDSL esterase/lipase [81], MLO protein [82], annexin [83], or protein SPO11-1 [47].

A meaningful meta-comparison, (H-/V-) vs. (V-/V+), shows how the genes influenced by infection in the sensitive cultivar are expressed in the resistant one. When overlaps of these datasets were investigated, totally fifteen DEGs were found (Table 2). All of them displayed opposite type of regulation in the two datasets. In other words, CMV infection induced in the cv. Vanda such changes that made the expression profile more similar to the cv. Heliana. Constitutive expression level of these genes in cv. Heliana (several of them are mentioned in the Section 2.3) may contribute to its resistant phenotype.

### 2.5. CMV-Inoculated Cultivars (Comparison H+/V+)

This comparison per se (Table S4) is less informative, in fact it is combination of previously mentioned comparisons. A meta-comparison (H-/V-) versus (H+/V+) is more interesting, as it reflects how the differences between CMV-free cultivars changed after their inoculation by CMV (Figure 1). A total of 34 of the 53 analyzed H-/V- DEGs did not significantly differ in the comparison H+/V+. Most of the remaining 19 DEGs belonged to the category with H+/V+ triplicate variance higher than 10%. Some of them, however, are mentioned in further text. Six DEGs showed equal type of difference between cultivars, but the expression difference was more intensive after inoculation. Three of them were more induced in the cv. Vanda—caffeoyl shikimate esterase (CSE), annexin and alanin aminotransferase (AlaAT).

CSE was constitutively prevalent in cv. Vanda (1.8-times over cv. Heliana) and after CMV challenge this difference increased to 4.8-fold. CSE is an enzyme essential for lignin biosynthesis, connected with growth, as well as with defense against pathogens [84,85].

Expression difference of annexin changed after inoculation from 2.4-fold to 5-fold. Annexins are group of proteins interacting with intracellular membranes and participating in organization of membrane-associated protein nets and relevant Ca2+-dependent signaling [86]. Expression or activity changes due to various types of abiotic and biotic stress may consist in their peroxidase activity or their function as signal molecules [87]. Thiel and Varrelmann [83] discovered interaction of filamentous annexin with the pathogenity factor P25 of beet necrotic yellow vein virus and hypothesized possible virus-targeted signal transfer in infected plants.

AlaAT is important for nitrogen and carbon metabolism in all living cells, especially at hypoxic stress [88], in some cases its expression has been correlated also with plant infection by viruses or cellular pathogens. It has been induced by powdery mildew in grapevine [89]. In pepper infected by different TMV pathotypes the AlaAT level was enhanced during incompatible interaction compared to compatible infection [90]. In our case there was an opposite situation (enhanced expression during compatible interaction), however, both experiments are not simply comparable (two host cultivars versus two virus pathotypes). Other authors found induction of AlaAT in *Arabidopsis* by tobacco rattle virus infection [91], which better corresponds to our data (1.5-times higher level changed after CMV infection to 2.1-fold).

Three DEGs were lower expressed in the susceptible cultivar and this difference even deepen after CMV challenge—genes for MRE11 protein, 7-etoxycoumarin-O-deetylase (ECOD) and protein from the family STRUBBELING-RECEPTOR 7.

MRE11 is repair protein of double-stranded breaks which acts by the mechanism of homologous recombination. Genome repair mechanisms are important for plant tolerance of biotic stress [92]. We recorded the change from 2.7 to 3.5-fold lower level in cv. Vanda.

ECOD (change from 1.7 to 2.5-fold lower level in cv. Vanda) is an oxidoreductase participating in monoterpenoid synthesis [93]. It is inducible by hydrogen peroxide during rooting [94]. No connection with pathogens or other type of stress has been observed till now.

STRUBBELING-RECEPTOR 7 family protein (change from 1.5 to 2-fold lower level in cv. Vanda) is potential R-protein with kinase activity. Its expression has been correlated positively with resistance of groundnut to tomato spotted wilt virus [95].

### 2.6. Context with Other Published Data

Comparison of transcriptomes of inoculated and control cucumber plants points the infection-induced and repressed genes. They code for both pro-viral factors helping the virus replication and anti-viral host defense factors. During the compatible response the expression of both gene groups oscillates. The balance shift in favor of anti-viral products and processes results in resistant phenotype. Several types of resistance (qualitative, quantitative, recessive or dominant genes-mediated) have been found in plants and particular genes have been mapped, e.g., RCY1 in *Arabidopsis* [96], Cmr1 in French bean [97], cmr2 in pepper [98], cmv1 in watermelon [99]. 

Biological function and mechanisms of action of these genes are still poorly understood. Jian et al. [100] discovered that the NO-producing nitrate reductase and an alternative oxidase pathway are important for the SA-mediated defense reaction against CMV in *A. thaliana*. In frame of a quantitative trait locus on the cucumber chromosome 6 ten candidate genes for CMV resistance were recently mapped [101]. They code for several RING finger proteins including E3 ubiquitin ligase, ethylene-sensitive and bZIP transcription factors and F box or LRR-domain containing proteins. According to our results, none of these specific genes showed significantly high expression in the resistant cv. Heliana, however, some functionally relative genes were found in positive correlation with published results (RING finger proteins with 1.5–2-fold lower expression in susceptible cv. Vanda). None of the relevant transcription factors clearly prevailed in any cultivar. It implicates potential differences in resistance genetics for various cucumber cultivars. On the other hand, considering the broad genetic and biological diversity within CMV [102], the specificity of virus isolate used in our experiment could also influence the obtained results.

CMV has an extremely broad host range, thus the reactions of different species to the virus challenge may differ considerably. Nevertheless, it is interesting that in frame of one biological species (*Cucumis sativus*) such dramatic difference between cultivars, i.e., high sensitivity and complete resistance to the same virus isolate was recorded. The majority of “omics“ works related to interaction with pathogens focus on the early infection states as the mechanisms leading to high resistance (hypersensitive reaction or immunity) must be initiated immediately after pathogen recognition. A detailed comparative analysis of CMV-induced tobacco transcriptome in different infection stages (6–20 dpi) showed that the number of DEGs more-or-less correlated with symptom manifestation [103]. Generally less data are for disposal from later stages of compatible and incompatible reactions. Therefore, we analyzed the plants 18 dpi when the infection of susceptible cultivar was well established with a high virus titer, while the resistant cv. Heliana showed no indicia of infection. Based on comparison of constitutive transcriptoms of the cultivars, potential resistance genes included especially PUX4, RIN4, MRE11 or nudix hydrolase 2. Further verification by independent biological experiments targeting these genes are needed. Thereafter, such genetic factors can be potentially applied in breeding programs for virus-resistant crops.

## 3. Materials and Methods

### 3.1. Virus and Plants

CMV isolate PK1 used in this study has been originally obtained from oilseed poppy (*Papaver somniferum*) plant [104]. The complete genome of RNA1, RNA2 and RNA3 segments (submitted to Genbank under accessions MN792886, MN792887 and MN792888, respectively) was obtained by high throughput sequencing of ribosomal-depleted total RNAs on an Illumina MiSeq platform (200-bp paired-end sequencing). Based on the Blast and phylogenetic analyses, the PK1 isolate is assigned to the subgroup II strain [102].

The cotyledons of *C. sativus* cv. Vanda and Heliana (Zelseed, Ltd.) were mechanically inoculated before true leaves development by the CMV isolate PK1 or by PBS solution (mock). The plants were cultivated under controlled insect-proof conditions (12 h light/12 h dark photoperiod, 55 μmol m^−2^ s^−1^ photon flux density, constant 22 °C temperature). Biological triplicates of the 2nd and 3rd true leaves were sampled 18 days post inoculation (dpi) and stored at −80 °C until analyzed. At this time the leaf symptoms (chlorotic spots) were fully developed in the case of infected cv. Vanda.

### 3.2. RNA Isolation, cDNA Library Preparation and Sequence Analysis

A total of 100 mg aliquots of stored leaf samples were ground and powdered using liquid nitrogen homogenization followed by total RNA extraction protocol using Spectrum Plant Total RNA Kit (Sigma). Extracted RNAs were quantified spectrophotometrically and 5 µg of total RNAs were used as input for ribosomal depletion reaction. Ribosomal RNAs were depleted using the Ribo-Zero rRNA Removal Kit (Illumina, San Diego, CA, USA). Freshly depleted RNA samples were fluorometrically quantified using Qubit™ RNA HS Assay Kit (Thermo Fisher Scientific, Waltham, MA, USA) and 40–65 ng were used for library preparation. RNA was fragmented 15 min at 94 °C and after reverse transcription the cDNA was used in PCR amplification with NEBNext Multiplex Oligos for Illumina (New England BioLabs, Ipswich, MA, USA) with 8 cycling steps. The final library was purified using NEBNext Sample Purification Beads. The concentration of samples was determined using Qubit™ dsDNA HS Assay Kit with Qubit Fluorometer v.2 (Thermo Fisher Scientific, Waltham, MA, USA) and fragment size assessed on Agilent 2100 Bioanalyzer (Agilent Technologies, Santa Clara, CA, USA). Finally. 2 × 150 paired end sequencing was performed using an Illumina NextSeq550 platform.

### 3.3. Data Analysis

Expression of individual genes from the cucumber genome (version Gy14 v2) [105] was estimated using Salmon (version 0.7.2) [106] for each sequenced sample separately. Count vectors were aggregated into the summary table and normalized for different sequencing depth between samples using edgeR (version 3.12.1) [107]. The tool was also used to assess the statistical significance of a change in expression between biological replicates of selected groups (H-, H+, V-, V+). We considered as significantly changed only transcripts that met 2 conditions; (1) the fold change between two conditions was at least 1.5; (2) the calculated false discovery rate was at most 0.05. GO annotations of those transcripts [108] were summarized with REVIGO [109] for more comprehensible visual inspection of affected functions. Data analysis processing was automated using pipelines implemented in the SnakeLines framework (manuscript in preparation) running on the Snakemake workflow engine (version 5.2.2) [110].

## Figures and Tables

**Table 1:** Number of differentially expressed genes (DEGs) in particular comparisons.

Comparison ^1^	Significant DEGs	Down-Regulated ^2^	Up-Regulated ^2^
H-/H+	9 (1)	8 (1)	1 (0)
V-/V+	3006 (359)	1379 (168)	1627 (191)
H-/V-	617 (53)	427 (32)	190 (21)
H+/V+	2456 (219)	1112 (99)	1344 (120)

^1^ H = Heliana, V = Vanda, - = mock-inoculated, + = CMV-inoculated; ^2^ Down- and up-regulation in sense “CMV-inoculated vs. mock” or “Vanda vs. Heliana”; Numbers of DEGs where values from biological triplicates differed by less than 10% are given in parentheses.

**Table 2:** Genes with opposite expression change in comparisons H-/V- and V-/V+.

ID	Function	Fold Change	
		H-/V-	V-/V+
CsGy7G013580.1	sodium/metabolite cotransporter BASS3, chloroplastic	−1.68	1.58
CsGy7G019210.1	pentatricopeptide repeat-containing protein At1g15510, chloroplastic	−1.62	1.71
CsGy6G013240.1	ubiquinone biosynthesis O-methyltransferase, mitochondrial	1.65	−2.06
CsGy4G018480.1	extradiol ring-cleavage dioxygenase	1.73	−1.53
CsGy2G023650.1	U-box domain-containing protein 12-like	1.83	−2.62
CsGy6G033990.1	epoxide hydrolase	1.93	−1.74
CsGy1G011590.1	sugar transporter, putative	2.29	−1.58
CsGy2G004500.1	26S proteasome regulatory subunit 6A homolog A	2.48	−2.11
CsGy7G018670.1	receptor like protein 9	2.57	−1.83
CsGy7G019140.1	serine/threonine-protein kinase receptor	3.35	−1.87
CsGy4G009730.1	receptor-like protein kinase FERONIA	4.02	−1.88
CsGy1G016880.1	sugar transporter, putative	4.88	−1.83
CsGy1G017330.1	sterile alpha motif, type 2	5.19	−2.82
CsGy4G004000.1	derlin	6.08	−1.83
CsGy4G018180.1	sigma factor binding protein 2, chloroplastic	9.84	−2.91

## Supplementary Materials

The following are available online at https://www.mdpi.com/2076-0817/9/2/145/s1, Table S1: Significant DEGs in cv. Heliana due CMV inoculation, Table S2: Significant DEGs in cv. Vanda due CMV inoculation, Table S3: Significant DEGs in mock-inoculated cvs. Vanda and Heliana, Table S4: Significant DEGs in CMV-inoculated cvs. Vanda and Heliana.
